# Association of the DASH dietary pattern with insulin resistance and diabetes in US Hispanic/Latino adults: results from the Hispanic Community Health Study/Study of Latinos (HCHS/SOL)

**DOI:** 10.1136/bmjdrc-2017-000402

**Published:** 2017-07-07

**Authors:** Leonor Corsino, Daniela Sotres-Alvarez, Nicole M Butera, Anna María Siega-Riz, Cristina Palacios, Cynthia M Pérez, Sandra S Albrecht, Rebecca A Espinoza Giacinto, Marisa Judith Perera, Linda Van Horn, M. Larissa Avilés-Santa

**Affiliations:** 1 Department of Medicine/Endocrinology, Duke University School of Medicine, Durham, North Carolina, USA; 2 Department of Biostatistics, University of North Carolina Health Care System, Chapel Hill, North Carolina, USA; 3 University of North Carolina Health Care System, Chapel Hill, North Carolina, USA; 4 Department of Public Health Sciences, University of Virginia School of Medicine, Charlottesville, Virginia, USA; 5 Department of Biostatistics and Epidemiology, Universidad de Puerto Rico Recinto de Ciencias Medicas, San Juan, Puerto Rico; 6 San Diego State University, San Diego, California, USA; 7 University of Miami School of Medicine, Miami, Florida, USA; 8 Northwestern University Feinberg School of Medicine, Chicago, Illinois, USA; 9 National Heart, Lung, and Blood Institute, Division of Cardiovascular Sciences, Bethesda, Maryland, USA

**Keywords:** DASH diet, diabetes, Hispanic, Latino, insulin resistance

## Abstract

**Objective:**

To examine the association between diet quality and both diabetes status and insulin resistance in Hispanic/Latino adults, and the extent to which differences in diet quality contribute to differences in outcomes across Hispanic/Latino heritage.

**Research design and methods:**

Cross-sectional study. Data are from 15 942 individuals enrolled in the Hispanic Community Health Study/Study of Latinos. Diet was ascertained using two 24-hour dietary recalls, and diet quality was measured using the Dietary Approaches to Stop Hypertension (DASH) score (range: 0–80, lowest to highest). Diabetes status was defined based on self-reported diagnosis, use of antihyperglycemic medications, or unrecognized diabetes (determined by baseline laboratory measures). Insulin resistance was determined using homeostatic model assessment of β-cell function and insulin resistance (HOMA-IR). The association between DASH and diabetes status was examined using multinomial logistic regression. The association between DASH and HOMA-IR was assessed using linear regression, and we tested whether the association was modified by Hispanic/Latino heritage or diabetes status.

**Results:**

DASH score was highest in those with self-reported diabetes (controlled) and no medications (44.8%). A higher DASH score was associated with a lower HOMA-IR, and the association was the same regardless of diabetes status (p>0.8 for the interaction).

**Conclusions:**

The association between DASH and diabetes status was strongest for those with controlled self-reported diabetes and who were not taking antihyperglycemic medications. A higher DASH score was associated with less insulin resistance among Hispanics/Latinos. Differences in DASH scores by Hispanic/Latino heritage did not explain the differences in prevalence of diabetes and insulin resistance reported in the diverse Hispanic/Latino population.

**Clinical trial number:**

NCT02060344

**Significance of this study**

**What is already known about this subject?**

- Dietary and lifestyle changes delay the onset of type 2 diabetes.
- The Dietary Approaches to Stop Hypertension (DASH) dietary pattern has also been shown to prevent type 2 diabetes because of its potential to improve insulin resistance and reduce hyperglycemia.

**What are the new findings?**

- The study reports a stronger association between DASH and controlled self-reported diabetes status and with participants who were not taking antihyperglycemic medications.
- A higher DASH score was associated with less insulin resistance among Hispanics/Latinos.
- Differences in DASH scores by Hispanic/Latino heritage did not explain the differences in prevalence of diabetes and insulin resistance reported in the diverse Hispanic/Latino population.

**How might these results change the focus of research or clinical practice?**

- A higher DASH score is indeed associated with lower insulin resistance. However, further research is needed in order to determine the role of different diet components and diabetes in the diverse Hispanic/Latino population of the USA.

## Introduction

Hispanics/Latinos represent 17.6% of the US population,^1^ and this figure continues to grow. The prevalence of type 2 diabetes mellitus among Hispanics/Latinos has been consistently higher than among non-Hispanic whites.^2^ In addition, the prevalence of diabetes mellitus has been shown to vary substantially among Hispanic/Latino heritage groups, from 10.2% in those of South American origin to 18% in those of Dominican, Puerto Rican, and Mexican origins.^3^ Similarly, there are significant differences in the prevalence of metabolic syndrome by Hispanic/Latino heritage.^4^ Although the specific reasons underlying these differences are unknown, they likely stem from a combination of genetic, biological, and cultural differences.^5^ Dietary behaviors, in particular, have been proposed as a major contributor in these disparities, since Hispanics/Latinos of diverse origins and heritages have different dietary patterns. In fact, our group recently reported significant variation in the consumption of food and macronutrients among Hispanic/Latino heritage groups.^6^

Previous research has demonstrated that dietary and lifestyle changes delay the onset of type 2 diabetes, and that certain eating habits may lead to changes in inflammatory markers and insulin resistance.^7–10^ The Dietary Approaches to Stop Hypertension (DASH) dietary pattern—which includes high intake of fruits, vegetables, whole grains, and low-fat dairy products—was originally developed to treat hypertension.^11 12^ An index using the DASH dietary pattern that scores various food and nutrient components is thus a measure of diet quality, with higher scores indicating a healthier diet. Following a DASH dietary pattern has also been shown to prevent type 2 diabetes because of its potential to improve insulin resistance and reduce hyperglycemia.^13 14^ A recent analysis based on the Insulin Resistance Atherosclerosis Study (IRAS, including 548 Hispanics/Latinos) showed an inverse association between adherence to the DASH dietary pattern and the incidence of type 2 diabetes.^14^ There are otherwise no studies that have examined the role of the DASH dietary pattern on diabetes and insulin resistance outcomes in a large and diverse Hispanic/Latino population.

Using baseline data from the Hispanic Community Health Study/Study of Latinos (HCHS/SOL), we examined the association between the DASH dietary pattern and type 2 diabetes and insulin resistance in Hispanic/Latino adults, and the extent to which differences in the DASH dietary pattern contribute to differences in diabetes status and insulin resistance across Hispanic/Latino heritage. Enhanced understanding of these associations in the diverse US Hispanic/Latino population may help prioritize the development of interventions targeting modifiable risk factors contributing to the risk for type 2 diabetes in this population.

## Research design and methods

The HCHS/SOL is a multicenter, prospective, population-based cohort study, and it is the largest epidemiologic study of Hispanics/Latinos in the USA. A total of 16 415 participants aged 18–74 years at screening from randomly selected households were recruited from four US locations: Bronx, NY; Chicago, IL; Miami, FL; and San Diego, CA. A detailed description of the HCHS/SOL sampling design and methods has been described elsewhere.^15 16^ In brief, the study was designed to include participants from Cuban, Dominican, Mexican, Puerto Rican, Central American, and South American heritages living in the selected communities, and adults aged 45–74 years were oversampled. All study participants provided informed consent, and the study had institutional review board approval from each institution participating in the study. For this analysis, we used baseline examination data (2008–2011).

## Study measurements and procedures

Enrolled participants completed a baseline examination in their preferred language (English or Spanish). All procedures and interviewer-administered questionnaires were conducted by centrally trained and certified bilingual study personnel following a standardized protocol, which included ongoing quality-assurance procedures. During the baseline visit, the following data were collected relevant to our research question and analysis: health behaviors such as dietary behaviors; medical history; and demographics, including age, sex, self-reported Hispanic/Latino heritage, years of education, and household income. Further, anthropometric measurements (including weight in kilogram, and height and waist circumference in centimeter) were performed by trained and certified staff following a standard protocol (www.cscc.unc.edu/hchs). Body mass index (BMI) was calculated as weight in kilogram divided by height in square meter. Blood samples were collected by a non-traumatic venous puncture after a fasting period of at least 8 hours prior to the visit. Participants with a fasting plasma glucose (FPG) <150 mg/dL and no previous diagnosis of diabetes completed a standard 75 g 2-hour oral glucose tolerance test (2hPG). Hemoglobin A1C and fasting insulin levels were collected. The assays’ methodologies and their procedures are described on the HCHS/SOL website (www.cscc.unc.edu/hchs).

## Outcomes

### Diabetes status

Diabetes status was defined from three main sources of information: self-reported physician-diagnosed diabetes, use of antihyperglycemic medications (scanned diabetes medications), and baseline laboratory collection (FPG, 2hPG, and A1C percentage). Specifically, participants were classified into one of the following seven mutually exclusive groups:

1. Normal glucose tolerance: FPG <100 mg/dL, 2hPG <140 mg/dL, A1C <5.7%, no history of diabetes, and not taking antihyperglycemic medications
2. Pre-diabetes FPG 100–125 mg/dL, or 2hPG 140–199 mg/dL, or A1C 5.7%–6.4%, and no history of diabetes and not taking antihyperglycemic medications

Participants with self-reported diabetes with optimal glycemic control (A1C <7%)

1. Taking antihyperglycemic medications
2. Not taking antihyperglycemic medications

Participants with self-reported diabetes without optimal glycemic control (A1C ≥7%)

1. Taking antihyperglycemic medications
2. Not taking antihyperglycemic medications
3. Unrecognized diabetes based on baseline laboratory collection:FPG ≥126 mg/dL, 2hPG ≥200 mg/dL, A1C ≥6.5%, and no self-reported history of diabetes, and not taking antihyperglycemic medications

We combined the seven-level diabetes status to create a two-level version of diabetes status (1—normal glucose or pre-diabetes, 2—diabetes) and a three-level version (1—normal glucose, 2—pre-diabetes, and 3—diabetes). Homeostatic model assessment of β-cell function and insulin resistance (*HOMA-IR*) was calculated for all participants as the product of fasting insulin (µU/mL) and fasting glucose (mmol/L) divided by 22.^17^

### DASH score

Dietary intake was assessed in all participants using two 24-hour recalls, one in person at the baseline visit and one via unannounced telephone call (30 days after the baseline visit, on average), using the multiple-pass methods of the Nutrition Data System for Research software, V.11, from the Nutrition Coordinating Center at the University of Minnesota. Recalls were excluded if energy intake was below the sequence (first or second)-sex-specific first percentile or above the 99th percentile, or if the recall was unreliable according to the interviewer. The DASH dietary pattern was scored based on the average of the two recalls using the components and standards for minimum and maximum scores from Günther *et al*.^18^ Briefly, the DASH score is the sum of eight component scores (grains, vegetables, fruits, dairy, red and processed meat, nuts/seeds/legumes, fats/oils, and sweets), each ranging from 0 (worst) to 10 (best). The grains component is the sum of the scores for the total grains and whole grains subcomponents, and the dairy component is the sum of the scores for total dairy and low-fat dairy subcomponents. Each of the four subcomponents ranges from 0 (worst) to 5 (best). DASH scores can range from 0 to 80. Higher DASH scores (healthier diet) indicate higher consumption of the grains, vegetables, fruits, dairy, and nuts/seeds/legumes components and lower consumption of the red and processed meat, fats/oils, and sweets components.

### Covariates

Participants reported their age, sex, Hispanic/Latino heritage, years of education (less than high school, high school, more than high school), annual household income (<$10 000, $10 000–$20 000, $20 000–$40 000, $40 000–$75 000, >$75 000, not reported), dietary acculturation, energy intake, current smoking status, and family history of diabetes. The first item of the dietary behavior questionnaire asked whether the participant’s foods are usually of Hispanic/Latino or American origin (dietary acculturation) using a five-level Likert scale (mainly Hispanic/Latino foods; mostly Hispanic/Latino foods and some American food; equal amounts of both Hispanic/Latino and American foods; mostly American foods and some Hispanic/Latino foods; and mainly American foods). For this analysis, we combined the ‘mainly’ and the ‘mostly’ categories, creating a three-level categorical variable. Energy intake (kcal) was calculated as the average energy (kcal) from both 24-hour dietary recalls.

### Statistical analyses

We excluded 473 participants due to either age >74 years at baseline (n=9) or missing DASH score (n=234), diabetes status (n=8), or HOMA-IR (n=222), yielding an analytical sample of 15 942 participants. There were no significant differences in baseline characteristics between participants included in the analysis versus those excluded due to missing data. HOMA-IR was log transformed before analyses. Distribution of demographic, health characteristics, and DASH dietary pattern is presented by diabetes status (seven mutually exclusive groups). In model 1, the association between DASH dietary pattern (score or tertiles) and diabetes status (two-level, three-level, and seven-level) was assessed using survey multinomial logistic regression adjusting by age, sex, Hispanic/Latino heritage, education, family income, family history of diabetes, smoking status, dietary acculturation, field center, and energy intake. Model 2 further adjusted by BMI and waist circumference, and model 3 added HOMA-IR. To test whether the association of DASH score and diabetes status differed by Hispanic/Latino heritage, we included the interaction between DASH and heritage. The association of DASH dietary pattern and HOMA-IR was assessed using linear regression adjusted by covariates specified in models 1 and 2 previously, and tested separately the interactions of DASH with seven-level diabetes status and with Hispanic/Latino background. When interactions were significant, at a 0.1 significance level, analyses were stratified; otherwise, models were reduced to exclude the interaction. All analyses accounted for the complex sample design and sampling weights using survey procedures in SAS V.9.3 and SAS-callable SUDAAN V.11.

## Results

In the target population, the average age was 41.1 years. Overall, 52.3% were female, the average weight was 78.9 kg, and 39.7% were obese. Table 1 provides demographic, diet, health characteristics, glucose, insulin, HOMA-IR, and mean DASH scores overall and by diabetes status. On average, those with diabetes (self-reported and unrecognized) were older and had a higher body weight, BMI, and HOMA-IR than those with normal glucose tolerance and pre-diabetes. HOMA-IR was highest in those with uncontrolled self-reported diabetes either taking or not taking antihyperglycemic medications (5.5 and 6.3, respectively). The mean DASH score was highest in those with self-reported diabetes controlled and no medications (44.8) followed by controlled and on medications (43.9), and the DASH score was lowest in those with normal glucose tolerance (41.3) and pre-diabetes (41.5). Those with uncontrolled self-reported diabetes and taking no medications had the highest consumption of total grains (score of 4.3). Those with uncontrolled self-reported diabetes taking medications had the highest consumption of vegetables (score of 4.6), and those with normal glucose tolerance had the lowest consumption (score of 4.1). Those with controlled self-reported diabetes on medications had the highest consumption of fruits (score of 4.3). Those with self-reported diabetes (both controlled and uncontrolled) and not taking medications also had the highest consumption of low-fat dairy (score of 3.1). Adults with self-reported diabetes controlled and not taking medications had the highest consumption of nuts, seeds, and dried beans (5.7), and they had the lowest (healthiest) consumption of meat, poultry, eggs, and fish (9.7); fats and oils (7.5); and sweets (1.7). Energy intake (kcal/day) was 300 kcal higher in those with normal glucose tolerance compared with those with self-reported diabetes taking medications (1625.6 kcal/day among those with uncontrolled diabetes and 1643.1 kcal/day among those with controlled diabetes) (table 1).

**Table 1:** Demographic, health, and DASH score by diabetes status, HCHS/SOL (2008–2011)

Characteristic	Overall (n=15 942)	Normal glucose tolerance (n=6590)	Pre-diabetes (n=6079)	SR diabetes controlled (medications) (n=706)	SR diabetes controlled (no medications) (n=255)	SR diabetes uncontrolled (medications) (n=1040)	SR diabetes uncontrolled (no medications) (n=171)	Unrecognized diabetes (n=1101)
Age (years)	41.1 (40.6, 41.6)	34.0 (33.5, 34.5)	45.2 (44.5, 45.8)	57.9 (56.6, 59.2)	51.5 (49.3, 53.7)	54.6 (53.3, 55.9)	49.0 (46.1, 52.0)	52.3 (51.0, 53.5)
Female	52.3 (51.2, 53.4)	54.5 (52.9, 56.1)	48.0 (46.0, 50.0)	56.5 (50.5, 62.3)	51.1 (41.7, 60.4)	55.7 (50.8, 60.5)	45.6 (35.2, 56.4)	56.5 (52.4, 60.5)
Diet acculturation								
Mainly/mostly Hispanic	73.6 (72.2, 75.0)	71.0 (69.2, 72.8)	75.2 (73.0, 77.3)	75.6 (70.5, 80.1)	80.5 (73.0, 86.3)	77.3 (73.2, 80.9)	83.6 (74.9, 89.8)	78.9 (74.7, 82.5)
Equally Hispanic/American	22.4 (21.1, 23.6)	24.4 (22.9, 26.0)	21.1 (19.2, 23.2)	21.0 (16.8, 25.9)	14.5 (9.7, 21.0)	19.7 (16.3, 23.6)	14.5 (8.9, 23.0)	18.3 (14.7, 22.4)
Mainly/mostly American	4.0 (3.6, 4.6)	4.6 (3.9, 5.4)	3.7 (2.9, 4.7)	3.4 (2.0, 5.6)	5.0 (2.2, 10.9)	3.0 (2.1, 4.4)	1.8 (0.5, 6.2)	2.9 (1.8, 4.5)
Current smoker	21.2 (20.0, 22.3)	21.6 (20.0, 23.2)	21.8 (20.2, 23.6)	14.9 (11.3, 19.4)	24.7 (17.1, 34.3)	19.0 (15.6, 23.0)	23.5 (15.6, 33.7)	17.3 (14.3, 20.8)
Weight (kg)	78.9 (78.4, 79.4)	75.3 (74.6, 76.0)	81.4 (80.6, 82.3)	85.2 (83.0, 87.3)	82.5 (79.5, 85.5)	85.0 (83.1, 86.9)	84.9 (81.0, 88.8)	83.5 (81.9, 85.1)
BMI (kg/m^2^)								
Underweight/normal	23.0 (21.9, 24.0)	33.0 (31.4, 34.7)	15.0 (13.6, 16.5)	9.7 (6.5, 14.3)	11.5 (7.3, 17.6)	9.4 (7.2, 12.2)	10.8 (5.5, 20.1)	8.7 (6.7, 11.3)
Overweight	37.3 (36.2, 38.5)	37.9 (36.2, 39.7)	38.3 (36.5, 40.2)	28.6 (23.9, 33.8)	35.3 (26.0, 45.9)	33.8 (29.7, 38.1)	37.3 (27.1, 48.7)	34.3 (30.5, 38.5)
Obese	39.7 (38.4, 41.0)	29.0 (27.2, 30.9)	46.7 (44.7, 48.6)	61.7 (56.0, 67.1)	53.2 (43.5, 62.6)	56.9 (52.2, 61.4)	51.9 (41.1, 62.5)	56.9 (52.9, 60.9)
Waist circumference (cm)	97.4 (97.0, 97.8)	93.1 (92.6, 93.7)	99.8 (99.2, 100.4)	106.3 (104.7, 107.9)	101.8 (99.4, 104.2)	107.0 (105.7, 108.4)	104.6 (101.6, 107.6)	104.2 (103.0, 105.3)
Fasting glucose (mmol/L)*	98.7 (98.2, 99.2)	89.5 (89.3, 89.7)	98.1 (97.8, 98.5)	111.8 (109.6, 114.0)	104.0 (100.6, 107.6)	170.5 (165.3, 175.9)	209.0 (191.6, 227.9)	124.2 (120.5, 128.0)
Fasting insulin (mU/L)*	10.2 (10.0, 10.5)	8.4 (8.1, 8.6)	11.9 (11.6, 12.2)	13.3 (12.3, 14.3)	12.1 (10.6, 13.8)	13.1 (12.2, 14.1)	12.2 (10.5, 14.2)	15.9 (15.1, 16.8)
HOMA-IR*	2.5 (2.4, 2.6)	1.8 (1.8, 1.9)	2.9 (2.8, 3.0)	3.7 (3.4, 4.0)	3.1 (2.7, 3.6)	5.5 (5.1, 6.0)	6.3 (5.5, 7.2)	4.9 (4.6, 5.2)
DASH score, 0–80	41.6 (41.3, 42.0)	41.3 (40.9, 41.7)	41.5 (41.0, 41.9)	43.9 (42.8, 44.9)	44.8 (43.1, 46.6)	42.6 (41.7, 43.6)	43.2 (40.8, 45.5)	42.5 (41.6, 43.4)
DASH tertiles								
Less healthy	35.9 (34.4, 37.3)	37.7 (35.8, 39.6)	35.4 (33.3, 37.6)	26.8 (22.6, 31.5)	25.5 (18.4, 34.1)	31.9 (27.6, 36.6)	31.2 (22.0, 42.2)	34.4 (30.5, 38.5)
Normal	33.7 (32.5, 34.8)	33.0 (31.5, 34.6)	35.5 (33.4, 37.6)	31.8 (27.2, 36.7)	33.8 (25.6, 43.0)	33.1 (28.1, 38.6)	31.1 (22.5, 41.2)	29.2 (25.8, 32.9)
Healthiest	30.5 (29.1, 31.9)	29.3 (27.5, 31.1)	29.1 (27.2, 31.0)	41.4 (35.6, 47.4)	40.8 (32.3, 49.9)	34.9 (30.5, 39.7)	37.7 (27.6, 49.0)	36.4 (32.3, 40.7)
Energy (kcal/day)	1914.4 (1893.9, 1934.8)	1958.1 (1930.8, 1985.5)	1943.1 (1908.5, 1977.7)	1643.1 (1558.5, 1727.8)	1822.0 (1672.0, 1971.9)	1625.6 (1566.9, 1684.3)	1895.3 (1740.5, 2050.2)	1771.8 (1710.8, 1832.9)

Data are presented as means or percentages (95% CIs).

*Geometric means are reported for variables with skewed distributions.

BMI, body mass index; DASH, Dietary Approaches to Stop Hypertension; HCHS/SOL, Hispanic Community Health Study/Study of Latinos; HOMA-IR, homeostatic model assessment of β-cell function and insulin resistance; SR, self-reported.

**Figure 1:**
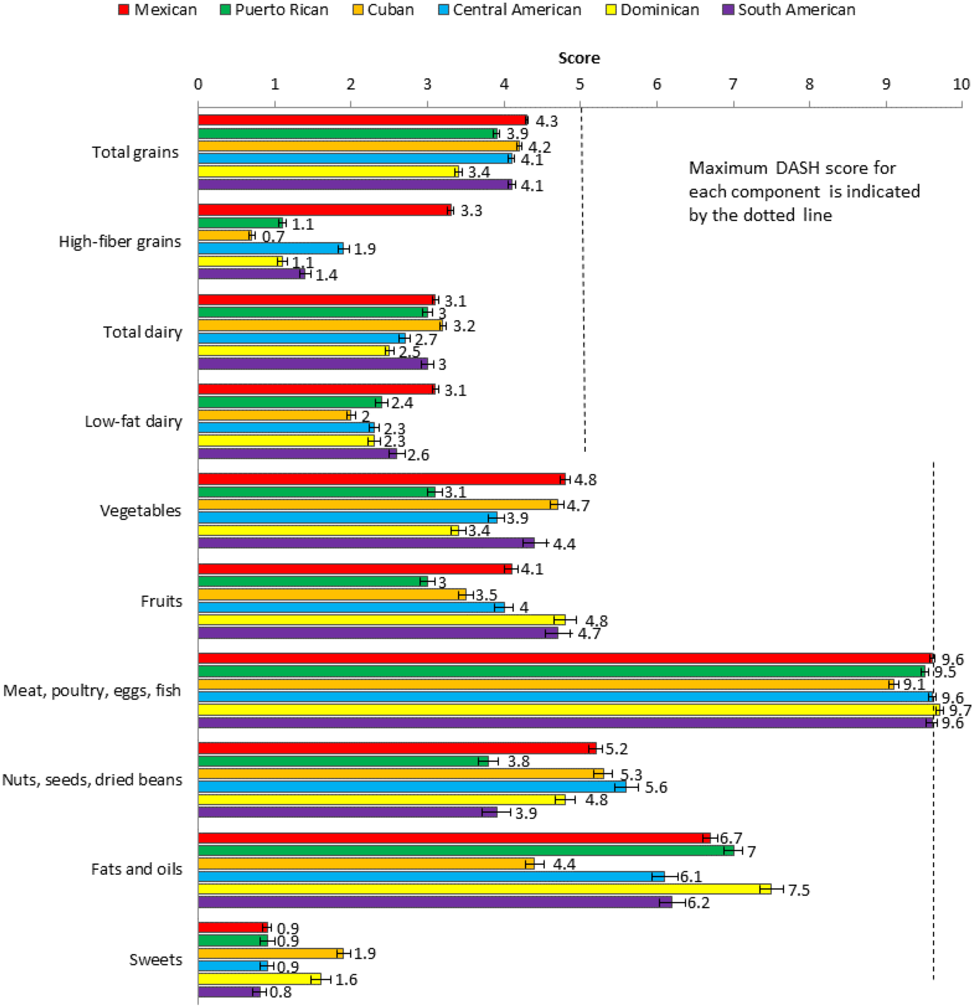
DASH component score by Hispanic/Latino heritage adjusted by age and sex. ^1^Adjusted by age and sex (mean age: 41.07, % male: 47.71). ^2^Each component ranges from 0 to 10, except for grains and dairy, for which each subcomponent ranges from 0 to 5. Data are presented as means±SE. DASH, Dietary Approaches to Stop Hypertension.

Figure 1 shows the mean DASH component scores by Hispanic/Latino heritage adjusted by age and sex. (In online supplementary figure, the model was further adjusted by diabetes status.) Overall, the mean DASH score was 41.6. Participants of Mexican descent had the highest mean score (45.0 (95% CI 44.7 to 45.4)), and those of Puerto Rican descent had the lowest (37.6 (95% CI 37.0 to 38.1)). Overall, the DASH food group with the healthiest scores was meat, poultry, eggs, and fish, with a mean score of 9.5 (95% CI 9.4 to 9.5); and the food group with the least healthy scores was sweets (mean score of 1.2 (95% CI 1.1 to 1.3) from a maximum score of 10). Those of Mexican descent had the highest score of total grains (mean 4.3 (95% CI 4.3 to 4.4) from a maximum of 5), vegetables (mean 4.8 (95% CI 4.7 to 5.0)), and low-fat dairy (mean 3.1 (95% CI 3.0 to 3.1) from a maximum of 5). Those of Cuban descent had the highest score of total dairy (mean 3.2 (95% CI 3.1 to 3.3)) and the highest score (lowest consumption) of sweets (mean 1.9 (95% CI 1.8 to 2.1) from a maximum of 5). Those of Central American descent had the highest score of nuts, seeds, and dried beans (mean 5.6 (95% CI 5.3 to 5.9)). Those of Dominican decent had the highest score of fruits (mean 4.8 (95% CI 4.5 to 5.1)) and the highest score (lowest consumption) of meats, poultry, eggs, and fish (mean 9.7 (95% CI 9.6 to 9.7)) and fats and oils (mean 7.5 (95% CI 7.2 to 7.8)). Those of South American descent had the lowest score (highest consumption) of sweets (mean 0.8 (95% CI 0.6 to 1.0)).

10.1136/bmjdrc-2017-000402.supp1 Supplementary data

### DASH score and diabetes status

Table 2 presents adjusted ORs and 95% CIs for the association of DASH dietary pattern (score and tertiles) with diabetes status. Interaction terms for DASH dietary pattern and Hispanic/Latino heritage were not significant; hence, results are pooled. After adjusting for heritage, age, sex, family history of diabetes, current smoking, dietary acculturation, education, income, field center, and energy intake, a higher DASH score was associated with diabetes (OR: 1.08 (95% CI 1.01 to 1.15)) self-reported and unrecognized combined. Further, a similar association was observed after adjusting for BMI and waist circumference (OR: 1.13 (95% CI 1.05 to 1.21)) and after adjusting for HOMA-IR (OR: 1.15 (95% CI 1.06 to 1.24)). However, when distinguishing among self-reported diabetes, whether controlled or not, and unrecognized diabetes, a higher DASH score was only associated with self-reported and controlled diabetes (OR: 1.18 (95% CI 1.02 to 1.36)), and it was higher among those not taking medications (OR: 1.40 (95% CI 1.15 to 1.69)).

**Table 2:** Adjusted ORs (95% CIs) for the association of DASH score (10-unit increment or tertiles) and diabetes status

Outcome levels compared		DASH (10 units)		DASH tertile			
				Medium versus low (less healthy)		High (healthiest) versus low (less healthy)	
		OR	95% CI	OR	95% CI	OR	95% CI
Diabetes (two levels)							
Diabetes versus pre-diabetes and normal glucose	Model 1	1.08	(1.01 to 1.15)	0.94	(0.79 to 1.11)	1.20	(1.02 to 1.41)
	Model 2	1.13	(1.05 to 1.21)	0.95	(0.80 to 1.13)	1.31	(1.11 to 1.54)
	Model 3	1.15	(1.06 to 1.24)	0.95	(0.79 to 1.14)	1.30	(1.08 to 1.57)
Diabetes (three levels)							
Pre-diabetes versus normal glucose	Model 1	0.92	(0.87 to 0.97)	1.02	(0.88 to 1.19)	0.84	(0.73 to 0.97)
	Model 2	0.95	(0.89 to 1.01)	1.03	(0.89 to 1.20)	0.90	(0.77 to 1.03)
	Model 3	0.95	(0.90 to 1.01)	1.01	(0.87 to 1.18)	0.89	(0.77 to 1.04)
Diabetes versus normal glucose	Model 1	1.02	(0.95 to 1.11)	0.95	(0.79 to 1.14)	1.08	(0.91 to 1.29)
	Model 2	1.09	(1.01 to 1.18)	0.97	(0.80 to 1.18)	1.22	(1.02 to 1.47)
	Model 3	1.11	(1.01 to 1.21)	0.95	(0.77 to 1.17)	1.20	(0.97 to 1.47)
Diabetes (seven levels)							
Pre-diabetes versus normal glucose	Model 1	0.92	(0.87 to 0.97)	1.02	(0.88 to 1.19)	0.84	(0.73 to 0.97)
	Model 2	0.95	(0.89 to 1.01)	1.03	(0.89 to 1.20)	0.90	(0.77 to 1.03)
	Model 3	0.95	(0.90 to 1.01)	1.01	(0.87 to 1.18)	0.89	(0.77 to 1.04)
Self-report controlled diabetes (medications) versus normal glucose	Model 1	1.09	(0.95 to 1.25)	1.09	(0.81 to 1.47)	1.34	(0.98 to 1.83)
	Model 2	1.17	(1.02 to 1.35)	1.12	(0.82 to 1.51)	1.55	(1.12 to 2.14)
	Model 3	1.18	(1.02 to 1.36)	1.08	(0.79 to 1.48)	1.50	(1.08 to 2.09)
Self-report controlled diabetes (no medications) versus normal glucose	Model 1	1.31	(1.09 to 1.58)	1.35	(0.82 to 2.22)	1.63	(1.03 to 2.59)
	Model 2	1.38	(1.14 to 1.66)	1.40	(0.84 to 2.31)	1.81	(1.14 to 2.88)
	Model 3	1.40	(1.15 to 1.69)	1.36	(0.81 to 2.29)	1.80	(1.11 to 2.90)
Self-report uncontrolled diabetes (medications) versus normal glucose	Model 1	1.00	(0.89 to 1.13)	1.00	(0.74 to 1.36)	1.05	(0.79 to 1.37)
	Model 2	1.08	(0.95 to 1.22)	1.04	(0.76 to 1.41)	1.19	(0.90 to 1.58)
	Model 3	1.10	(0.96 to 1.25)	1.02	(0.73 to 1.42)	1.18	(0.87 to 1.60)
elf-report uncontrolled diabetes (no medications) versus normal glucose	Model 1	1.02	(0.78 to 1.34)	0.92	(0.52 to 1.62)	1.02	(0.54 to 1.91)
	Model 2	1.08	(0.82 to 1.43)	0.95	(0.53 to 1.69)	1.15	(0.60 to 2.19)
	Model 3	1.13	(0.85 to 1.50)	0.94	(0.52 to 1.71)	1.17	(0.61 to 2.26)
Unrecognized diabetes versus normal glucose	Model 1	0.95	(0.85 to 1.06)	0.79	(0.63 to 0.99)	0.93	(0.73 to 1.19)
	Model 2	1.00	(0.90 to 1.12)	0.80	(0.64 to 1.01)	1.03	(0.81 to 1.32)
	Model 3	1.00	(0.89 to 1.13)	0.77	(0.60 to 0.98)	0.98	(0.75 to 1.27)

Model 1: DASH + heritage + age + sex + diabetes family history + current smoker + dietary acculturation + education + income + field center + energy intake.

Model 2: Model 1 + body mass index + waist circumference.

Model 3: Model 2 + HOMA-IR.

DASH, Dietary Approaches to Stop Hypertension; HOMA-IR, homeostatic model assessment of β-cell function and insulin resistance.

### DASH dietary pattern and HOMA-IR

Table 3 presents adjusted difference in HOMA-IR by DASH score. Interaction terms for DASH dietary pattern (score or tertiles) with seven-level diabetes status (p>0.8) and with Hispanic/Latino background were not significant (p>0.1). Hence, results were not stratified. A 10-unit higher DASH score was associated with a 4% lower HOMA-IR (95% CI ^-^5.97%–^-^2.06%) after adjusting for diabetes status, family history of diabetes, heritage background, age, sex, education, income, current smoking, dietary acculturation, field center, and energy intake. After adjusting for BMI and waist circumference, a 10-unit higher DASH score was associated with a 1.74% lower HOMA-IR (95% CI ^-^3.34%–^-^0.14%). The lower HOMA-IR was slightly larger (6%) among adults in the high DASH tertile (healthier diet) than among those in the lowest tertile (less healthy diet).

**Table 3:** Adjusted difference in HOMA-IR by DASH score

	DASH (10 units)		DASH tertile			
			Medium versus low (less healthy)		High (healthiest) versus low (less healthy)	
	% Change*	95% CI	% Change*	95% CI	% Change*	95% CI
Model 1	−4.01	(−5.97 to 2.06)	0.96	(−2.72 to 4.65)	−5.96	(−10.48 to 1.43)
Model 2	−1.74	(−3.34 to 0.14)	1.21	(−1.92 to 4.34)	−1.57	(−5.32 to 2.19)

Model 1: DASH + Hispanic/Latino heritage + age + sex + diabetes family history + current smoker + dietary acculturation + education + income + field center + energy intake + diabetes status (seven levels).

Model 2: Model 1 + body mass index + waist circumference.

*Percent change is calculated as 100×β estimate.

DASH, Dietary Approaches to Stop Hypertension; HOMA-IR, homeostatic model assessment of β-cell function and insulin resistance.

## Discussion

To our knowledge, the current study is the first to show an association between adherence to the DASH dietary pattern and diabetes status and insulin resistance among the diverse Hispanic/Latino adult population in this country. The DASH dietary pattern has been considered one of the best eating plans in consecutive years and is currently one of the recommended diets for the management of hypertension.^19–21^ The benefits of the DASH dietary pattern have been documented by several trials in the management of patients with hypertension and for its benefits for inflammatory markers, insulin resistance, and diabetes.^8–10^

An analysis based on the IRAS (which included 548 Latinos) demonstrated an inverse association between adherence to the DASH dietary pattern and the incidence of type 2 diabetes after 5 years of follow-up.^14^ In our cross-sectional analysis, we showed a positive association between DASH score and diabetes, with a stronger association among those who self-reported diabetes and had it controlled without medications. It is to be expected that adults with self-reported diabetes might be more likely to follow dietary recommendations from their providers and more likely to change their eating habits after being diagnosed with diabetes. We previously published data showing that patients with a pre-existing diagnosis of diabetes and/or hypertension were more likely to report receiving lifestyle behavior recommendations from their providers compared with those without diabetes or hypertension.^22^ Despite the higher prevalence of diabetes among Hispanics/Latinos of Mexican, Puerto Rican, and Dominican heritages in our study, we also observed a higher DASH score among those of Mexican-American heritage and the lowest score in those of Puerto Rican heritage, despite both groups having a high prevalence of diabetes. This discrepancy may be explained in part due to a difference in the consumption of different components of the DASH diet and not only due to the overall score. For example, Mexican-Americans reported a higher consumption of grains, vegetables, and low-fat dairy, whereas Puerto Ricans reported lower consumption of high-fiber grains and vegetables and lower (healthier) consumption of fat and oils. The main reason for this difference is more complex since our data also showed Cuban-Americans—one of the groups with the lowest prevalence of diabetes—reported a healthier mean consumption of sweets; total dairy; and nuts, seeds, and dry beans. Similarly, participants of South American heritage reported a higher consumption of sweets. Thus, the pathophysiology of diabetes and insulin resistance is very complex and has multiple contributing factors. Diet plays a significant role, but in the case of Hispanics/Latinos, it might not explain the reported difference in prevalence in diabetes and insulin resistance within this population. Our results, however, confirm previously reported findings that a higher DASH score is associated with a lower insulin resistance (HOMA-IR), in the same way in which the DASH dietary pattern has been associated with decreased levels of insulin resistance and inflammatory markers.^13 14^

Our findings should be considered in light of the following limitations. First, the cross-sectional design of the study precludes causal conclusions. Second, the dietary information is based on self-reported data and is subject to recall and social desirability biases.^23 24^ However, this study provides valuable new information about associations between the DASH dietary pattern and diabetes and insulin resistance among the large and diverse Hispanic/Latino population in the USA.

## Conclusions

In the largest epidemiologic study ever conducted in the USA with a diverse Hispanic/Latino population, participants with self-reported diagnosis of diabetes and unrecognized diabetes reported the highest DASH score. Differences in the DASH score do not completely explain the differences in the prevalence of diabetes within Hispanics/Latinos from different heritage backgrounds. Further, we confirm that a higher DASH score is indeed associated with lower insulin resistance, as previously reported in other segments of the US population. Future research is needed to further elucidate the role of different diet components and diabetes in the Hispanic/Latino population.

10.1136/bmjdrc-2017-000402.supp2 Supplementary data
