# Supplementary figures and images for: Association of the DASH dietary pattern with insulin resistance and diabetes in US Hispanic/Latino adults: results from the Hispanic Community Health Study/Study of Latinos (HCHS/SOL)
Source: BMJ Open Diabetes Res Care. 2017 Jul 7;5(1):e000402. doi: 10.1136/bmjdrc-2017-000402 (PMC5530245; doi:10.1136/bmjdrc-2017-000402)

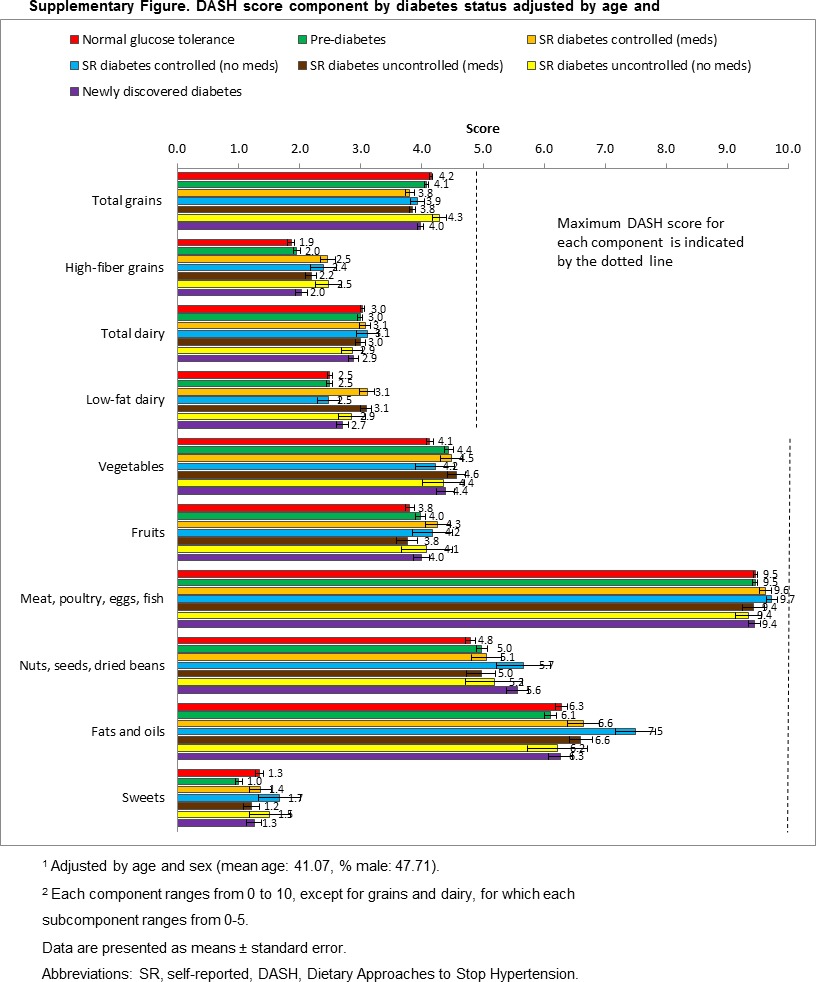

Supplement: Supplementary data [file bmjdrc-2017-000402supp002.jpg]
