# Supplementary material for: Association of the DASH dietary pattern with insulin resistance and diabetes in US Hispanic/Latino adults: results from the Hispanic Community Health Study/Study of Latinos (HCHS/SOL)
Source: BMJ Open Diabetes Res Care. 2017 Jul 7;5(1):e000402. doi: 10.1136/bmjdrc-2017-000402 (PMC5530245; doi:10.1136/bmjdrc-2017-000402)
Supplement: Supplementary data [file bmjdrc-2017-000402supp001.docx]

Department of Medicine

Division of Endocrinology, Metabolism and Nutrition

April 20, 2017

Guillermo Umpierrez, MD, CDE, FACE, FACP
Editor in Chief
BMJ Open Diabetes Research & Care

Ref: bmjdrc-2017-000402 - "Association of the DASH dietary pattern with insulin resistance and diabetes in U.S. Hispanic/Latino adults: results from the Hispanic Community Health Study/Study of Latinos (HCHS/SOL)

Dear Dr. Umpierrez and the BMJ Open Diabetes Research and Care Editorial Board and Reviewers.

On behalf of my co-authors, I submit the revise version of our manuscript. Our group has addressed all the questions asked by the editors and reviewers. All changes are highlighted in the manuscript using yellow text. Responses can be find in the authors response section of the online manuscript submission site.

Thank you for your consideration of our manuscript. Please feel free to contact me by Email with any questions or notifications.

Sincerely yours,

*Leonor Corsino, MD, MHS, FACE*

Assistant Professor of Medicine

Department of Medicine, Division of Endocrinology, Metabolism, and Nutrition.

DUMC Box 3451, Durham, NC

Phone: 919-668-1367

Fax: 919-668-1366

Email: [corsi002@mc.duke.edu](mailto:corsi002@mc.duke.edu); [leonor.corsinonunez@duke.edu](mailto:leonor.corsinonunez@duke.edu)
